# A Cerebellar Partitioning Method Using Spectral Clustering With Optimized Nonlinear Functional Connectivity

**DOI:** 10.1002/hbm.70268

**Published:** 2025-07-01

**Authors:** Tengyue Wang, Kai Zhou, Xiaoyan Zhou, Xiaoming Wang, Haoyang Xing, Rong Li, Wei Liao, Jiali Yu, Fengmei Lu, Xiaofei Hu, Huafu Chen, Qing Gao

**Affiliations:** ^1^ School of Mathematical Sciences The Clinical Hospital of Chengdu Brain Science Institute, MOE Key Laboratory for Neuroinformation, University of Electronic Science and Technology of China Chengdu People's Republic of China; ^2^ Health Management Center, Department of Neurology Affiliated Hospital of North Sichuan Medical College Nanchong People's Republic of China; ^3^ Huaxi MR Research Center (HMRRC), Department of Radiology, Huaxi Hospital, College of Physics, Sichuan University Chengdu People's Republic of China; ^4^ The Clinical Hospital of Chengdu Brain Science Institute, MOE Key Laboratory for Neuroinformation, High‐Field Magnetic Resonance Brain Imaging Key Laboratory of Sichuan Province, School of Life Science and Technology University of Electronic Science and Technology of China Chengdu People's Republic of China; ^5^ The Department of Radiology Southwest Hospital, Third Military Medical University Chongqing People's Republic of China

**Keywords:** cerebellar partitioning, clustering ensemble, connectivity matrix, functional magnetic resonance, spectral clustering algorithm

## Abstract

Cerebellum has a stronger individual specificity of functional signals than the brain and is associated with a variety of neuropsychiatric disorders, and increasing attention is being paid to neuropsychiatric symptoms caused by cerebellar dysfunction. However, there is a lack of a suitable cerebellar partition utilizing researchers to fully understand the functional and structural organization of the cerebellum, reduce data dimensionality, and improve the applicability of various types of models to cerebellar functional imaging data, impeding progress in cerebellum‐related research. In this study, we use order‐preserving variations with spatial constraints to optimize functional connectivity matrices and employ a spectral clustering algorithm combined with a clustering ensemble technique to construct a cerebellar partitioning algorithm with a variable number of partitions. Our method was initially validated by using two separate sets of functional magnetic resonance data (fMRI), demonstrating high reproducibility across individuals. Comparative analysis revealed that our partitions exhibited enhanced signal coherence and greater spatial congruence with established cerebellar structural templates compared to four publicly available cerebellar atlases. Furthermore, preliminarily applying these partitions to Parkinson's disease (PD) data, we extracted cerebellar connectivity network features and constructed a classification model using a logistic regression model with L2 regularization. The connectivity features derived from our newly constructed cerebellar partitions substantially improved the usability of the Parkinson's classification model, with the classification of PD optimized at a number of partitions equal to 185, suggesting that the optimal number of cerebellar partitions may also vary based on the problem under study. Notably, cerebellar regions implicated in motor execution were identified to exhibit higher feature importance in the Parkinson's classification model, offering an important direction for feature selection in the multimodal classification models of PD.

## Graphical abstract

By incorporating nonlinear order‐preserving transformations and spatial constraints to optimize the distribution of functional connectivity (FC) in the cerebellum, the cerebellar partitioning obtained using spectral clustering provides stable and reliable results in terms of reproducibility, spatial continuity, and signal homogeneity. The features extracted using this cerebellar partitioning can also be used to effectively identify Parkinson's disease (PD).

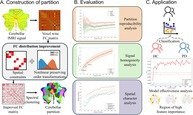

## 1 Introduction

The human brain exhibits a dual nature of functional segregation and integration, which is crucial to conceptualize and model in the analysis of brain functional networks. Accurate brain partitioning is essential for exploring the brain functional structure, encompassing both segregation and integration, to understand the neural mechanism of cognition and behavior. A most common approach for delineating functional segregation is the voxel‐based partitioning of brain regions, which offers the most detailed model but suffers from computational burdens, high susceptibility to noise, and limited interpretability (Thirion et al. 2014). Furthermore, voxel‐scale data often contains redundant information, which can be leveraged to substantially reduce the dimensionality of functional magnetic resonance imaging (fMRI) datasets (Craddock et al. 2011). Another prevalent method for functional segregation involves the use of independent component analysis (ICA) to isolate significant components from the overall independent components, guided by peak coordinates and temporal characteristics. While this approach effectively reduces data dimensionality, the resulting network distribution tends to be more discrete, and potential discrepancies in data resolution or preprocessing may lead to the omission of crucial cortical topographical features (Thomas Yeo et al. 2011). The optimal way to functional brain partitioning subject to specific research objective remains an active area of investigation and refinement, necessitating ongoing exploration and innovation in the field.

Over the past few decades, a wealth of scholarly inquiry has been directed toward the cerebral partitioning, with a particular emphasis on the application of resting‐state fMRI (rs‐fMRI). Two primary approaches have emerged in this domain: the local gradient approach and the global similarity approach (Schaefer et al. 2018). The global similarity approach is fundamentally rooted in the clustering of all voxels based on their connectivity profile similarity (Li et al. 2023). In the realm of global similarity‐based brain partitioning studies, spectral clustering techniques have gained prominence (Arslan et al. 2018; Bajada et al. 2020). In addition, gradient‐based methodologies define region boundaries by detecting abrupt changes in local connectivity, a process known as boundary mapping. This approach hinges on the identification of sudden transitions in resting‐state functional connectivity (RSFC) from one spatial location to a neighboring location (Eickhoff et al. 2018). These transitions are discerned by calculating the local gradient across the entire RSFC pattern of the brain (Gordon et al. 2016). Notably, Schaefer's work combines elements of both global similarity and local gradients to propose the gradient‐weighted Markov Random Field model for brain partitioning (Schaefer et al. 2018). This model has been notably applied in the research by Liu et al. (2023), where the brain is partitioned into a continuum of 100 to 1000 ROIs, with the resultant maps being utilized for functional connectivity network analysis.

However, compared to the cerebral cortex, the cerebellum has been relatively poorly partitioned despite its comparable diversity, complexity, and pronounced individual variability (Marek et al. 2018; Ren et al. 2019). To comprehend the similarities and distinctions between the cerebellum and the cerebral cortex, a comprehensive functional compartmentalization of the cerebellum is essential. Conventional cerebellar atlases such as Anatomical Automatic Labeling (AAL) and probabilistically optimized spatially unbiased infra‐tentorial template (SUIT) offer structural delineations but fall short in capturing the functional distribution of the cerebellum (Diedrichsen and Zotow 2015; Rolls et al. 2020), but such structural partitions cannot fully reflect the functional distribution and connectivity of the cerebellum, and with the large differences in the volume of different structures of the cerebellum, its small‐scale properties are more susceptible to interference by noise signals when utilizing the two templates mentioned above. Some scholars have undertaken task‐driven cerebellar partitioning. For instance, King employed non‐negative matrix decomposition to establish a Multi‐Domain Task Battery (MDTB) atlas, dividing the cerebellum into ten partitions based on task‐state fMRI data (King et al. 2019). Buckner devised templates for 7‐network and 17‐network cerebellar partitioning by correlating the cerebellar cortex with corresponding brain sub‐network divisions (Buckner et al. 2011). All of the above methods divide the cerebellum into a defined number of partitions with a small number of partitions. Although a smaller number of partitions can improve computational efficiency, their resolution and homogeneity may not achieve the researcher's goal. For example, studying more detailed functions requires finer cerebellar partitions (Nettekoven et al. 2024). Some researchers have also pointed out that most cerebellar partitions with fewer than 50 regions do not provide a better picture of the functional diversity of the cerebellum (Ren et al. 2019). Therefore, there is a need to provide different scales of cerebellar partitioning based on different research purposes, while cerebellar partitioning based on graphical model clustering method can change the scale of partitioning by adjusting the number of clusters. Established studies have tended to disregard the cerebellum's distinctive specificity and exhibit limited result stability (Ji et al. 2019; Ren et al. 2019). The utility of these partitions as nodes in cerebellar network analyses is still a matter of debate. Consequently, an accurate, stable, and appropriately scaled cerebellar partitioning method is imperative to serve as a foundation for precise cerebellar dimensionality reduction, thereby deepening our comprehension of the individual characteristics of the cerebellum. In the present study, we introduced a data‐driven approach for cerebellar partitioning and assessed its reliability against the four established criteria (Arslan et al. 2018; Bryce et al. 2021; Craddock et al. 2011). The process began with the application of spatial constraints to sparsify the FC matrix, followed by distinct order‐preserving mappings of the sparsified FC matrix. Subsequently, we employed the processed FC matrix as a distance metric and utilized spectral clustering for the partitioning of the cerebellum (Bajada et al. 2020; Craddock et al. 2011; Seghier 2018). In parallel, group‐level cerebellar partitions were constructed using an ensemble clustering approach (Craddock et al. 2011; Nikolaidis et al. 2020). Regional continuity was ensured by the construction of spatially constrained similarity matrices. To validate the reproducibility and regional continuity of this novel partitioning method, the performance of the partitions across two independent datasets were compared. Moreover, we substantiated adherence to the other two delineated criteria by comparing our constructed cerebellar partition with several established cerebellar templates. We extended the application of this partitioning approach to two distinct datasets: the Human Connectome Project (HCP) and the Southwest Hospital dataset. To further explore the possible implications of cerebellar partitioning, we applied the model to the classification and identification of Parkinson's disease (PD), as well as the delineation of key regions, thereby enhancing our understanding of the method's utility in clinical contexts.

## 2 Materials and Methods

### 2.1 Overview

**FIGURE 1:**
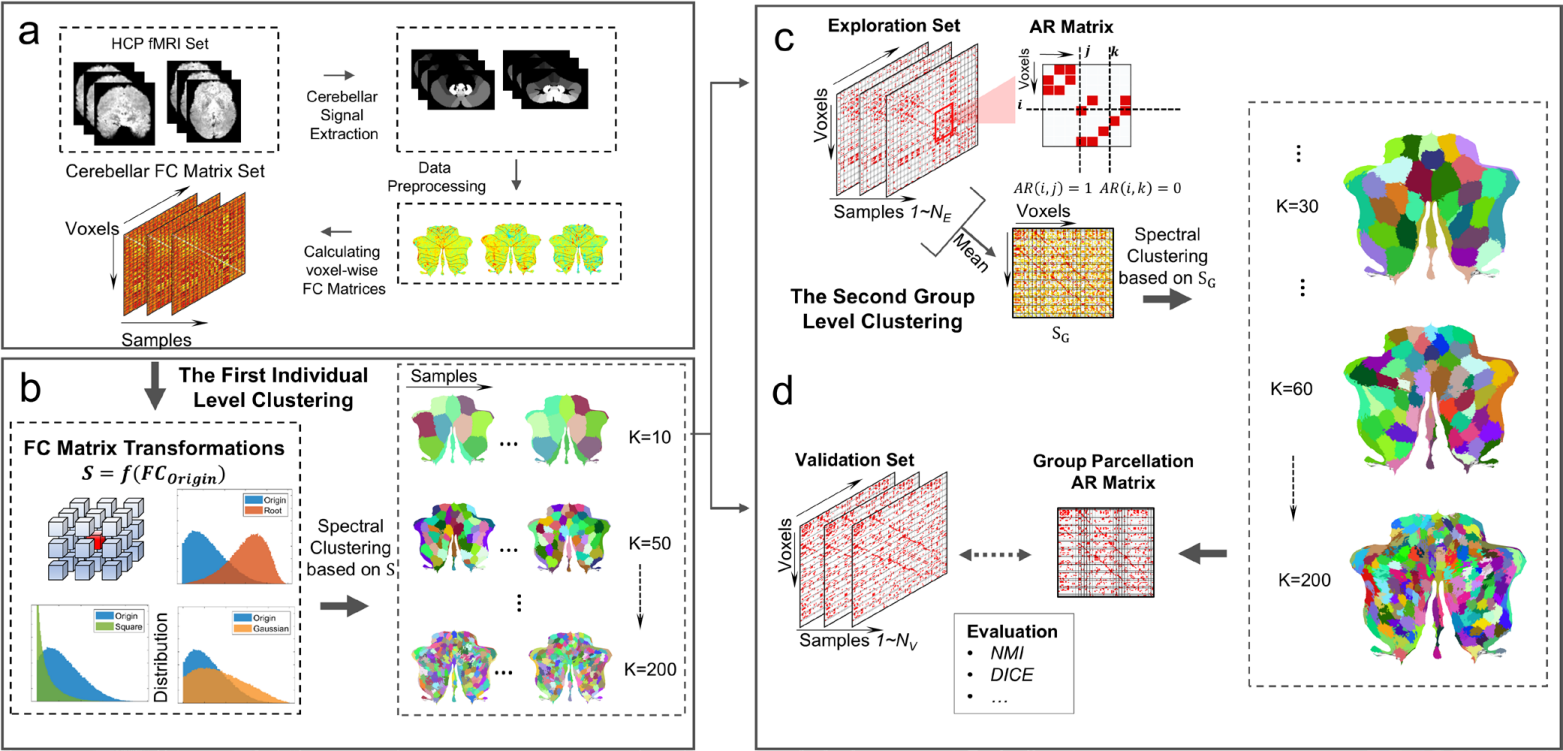
Group level partitioning data processing, algorithm construction and evaluation process. (a) The extraction of cerebellar voxel signals and the computation of their functional connectivity matrices; (b) the functional cerebellar partitioning of the algorithm at the individual level; (c) the group level cerebellar partitioning constructed based on the idea of cluster ensemble; (d) the reproducibility study of the algorithm on the validation set.

We summarized and outlined the logical relationships between data preprocessing and each subsequent step of the algorithm. The complete workflow, encompassing data processing, algorithm development, and evaluation, is visually represented in Figure 1, offering a clear overview of the methodological progression.

The first step of the process involves data extraction, as depicted in Figure 1a. Firstly, all sample data were processed one by one, including three steps: data preprocessing, cerebellar signal extraction, and the construction of the FC matrix. In the second step, all individual data were segmented, delineated in Figure 1b, and four similarity matrices denoted as *S* are constructed based on the FC matrices of each sample. Then, based on different measurement matrices, the first individual clustering was carried out for all samples, and the number of clusters gradually increases from *K* = 10 to 200 with an increment step of 5. This produced a total of 4 × 39 sets of clustered sample results as illustrated in Figure 1b.

The third step of the process, outlined in Figure 1c, shows the construction process of group‐level partition. Each sample set of clustering results was divided into an exploration set (57 participants) and validation set (25 participants) in a ratio of 7:3. All clustering result vectors based on the second step are transformed into adjacency representation (AR) matrices. The averaged AR matrix of all participants in the exploration set was taken as the similarity metric matrix *S*_*G*_ of group‐level clustering. Based on the similarity metric matrix *S*_*G*_ of group level clustering, spectral clustering algorithm was applied to complete the construction of 39 group‐level clustering partitioning results, with the number of partitions ranging from 10 to 200.

In the evaluation stage of the process (Figure 1d), the group‐level clustering results were represented by AR matrix and compared with the AR matrix of the validation set. The NMI and Dice coefficients were calculated as evaluation criteria for method reproducibility. The partitions with the best performance will be compared with the four public templates, SUIT, MDTB, Buckner and Pezoulas for signal consistency. Finally, the optimal partition was selected and the similarity with the four public templates is calculated to explore the interpretability and characteristics of the newly constructed partition.

This comprehensive procedure was subsequently replicated utilizing the Southwest Hospital dataset and the resulting constructed cerebellar partitions were applied to a subsequent PD classification task.

### 2.2 Materials and Data Preprocessing

One dataset in this study includes 82 participants of publicly available fMRI data from the Human Connectome Project (HCP) 100 unrelated dataset with the criteria that respiratory, cardiac, and head movement data are available in all runs (rfMRI_REST1_LR, rfMRI_REST1_RL, rfMRI_REST2_LR, and rfMRI_REST2_RL). REST1 and REST2 were acquired on two different days. This dataset excludes the effect due to the covariate of household (Amico and Goñi 2018). The HCP scanning protocol was approved by the local Institutional Review Board at Washington University in St. Louis. Informed consent was obtained from all subjects (Wang et al. 2018). The images were acquired using an echo planar imaging (EPI) sequence, capturing a total of 1200 time points with a temporal resolution of 0.72 s. The voxel size is 2 × 2 × 2 mm^3^, while the data space dimensions are 91 × 109 × 91. Flip angle (FA) was set to 52° and the field‐of‐view (FOV) was 208 × 180 mm (RO × RE). Additionally, each layer thickness was 2 mm, with a total of 72 layers scanned. For an exhaustive list of scan parameters and additional technical details, please refer to the link provided: https://www.humanconnectome.org/storage/app/media/documentation/s1200/HCP_S1200_Release_Appendix_I.pdf.

For the HCP dataset, the minimal preprocessing pipeline was first utilized. This involved B0 distortion correction, head motion correction, structural registration, normalization of all participant brain images to a 4D averaged brain template, and non‐linear transformation of the data to Montreal Neurological Institute (MNI) space (Glasser et al. 2013). Afterward, covariate regression was performed to mitigate the impact of data's heartbeat signal noise, and a bandpass filter was applied, extracting signals within the range of 0.01–0.1 Hz. It is important to note that although spatial smoothing of the whole‐brain signal during data preprocessing can enhance the signal‐to‐noise ratio, it artificially elevates the spatial correlation between local regions, which significantly affects subsequent calculations. Thus, spatial smoothing processing was avoided during this data preprocessing step. Finally, the fMRI data signals from the cerebellar gray matter in the lobule and vermis regions were extracted from the whole brain using the SUIT cerebellar structure template. All data processing operations were done on The Data Processing & Analysis for Brain Imaging (DPABI) 6.0 software (Yan et al. 2016).

Another dataset contains fMRI data for 154 patients with PD from Southwest Hospital in Chongqing, China, as well as 109 healthy control (HC) participants with matched age and sex. Meanwhile, the Minimum Mental State Examination (MMSE), Montreal Cognitive Assessment (MOCA) measuring cognitive level, and the Hamilton Depression Scale (HAMD) measuring depression level were collected for PD. This study was approved by the Medical Ethics Committee of the Southwest Hospital (Chongqing, China), and written informed consent was obtained from all participants before enrollment. Detailed information on the demographic and clinical characteristics for the two groups is shown in Table 1.

**TABLE 1:** Demographic and clinical characteristics.

	Parkinson's disease (PD)	Healthy control (HC)
Size	154	109
Sex (male/female)^a^	77/77	36/73
Age (mean ± Std)^b^	61.63 ± 9.61	56.56 ± 6.07
Course of disease (mean ± Std)	6.62 ± 4.69	
Start age (mean ± Std)	55.01 ± 10.94	
MMSE (mean ± Std)	26.51 ± 3.72	
MOCA (mean ± Std)	21.4 ± 5.22	
HAMD (mean ± Std)	15.35 ± 7.55	

^a^ *χ* ^2^ = 6.826, *p* < 0.05, chi‐square test.

^b^ *t* = 4.573, *p* < 0.05, two‐sample *t* test.

All MRI data of the participants were acquired using a 3.0 T MRI scanner (TIM Trio, Siemens, Erlangen, Germany) equipped with an 8‐channel phased‐array head coil. Resting‐state fMRI was obtained through an EPI sequence and 240 time points were collected. The following scan parameters were used: 36 slices, no gap, sequential acquisition, voxel size of 3 × 3 × 3.99 mm^3^, a repetition time (TR) of 2000 ms, an echo time (TE) of 30 ms, FA of 90°, FOV of 256 × 256 mm^2^, and matrix size of 64 × 64. Data preprocessing on this dataset is consistent with the HCP dataset.

### 2.3 Similarity Measurement Optimization

In the functional brain partitioning studies, functional connectivity strength is commonly utilized as the similarity measure criterion for clustering algorithms. This metric is defined by the Pearson correlation coefficient value between the time series of two voxels:

(1) $${\mathrm{FC}}_{\mathrm{ij}}=\frac{\sum_{t=1}^{T}\left(x_{t}-\bar{x}\right)\left(y_{t}-\bar{y}\right)}{\sqrt{\sum_{t=1}^{T}{\left(x_{t}-\bar{x}\right)}^{2}\sum_{t=1}^{T}{\left(y_{t}-\bar{y}\right)}^{2}}}$$

where *T* is the time series length, $x_{t}$ denotes the signal value of the voxel *i* at the time *t*, $y_{t}$ denotes the signal value of the voxel *j* at the time *t*, $\bar{x}$ denotes the mean of $x_{t}$, $\bar{y}$ denotes the mean of $y_{t}$.

However, the magnitude of Pearson's correlation coefficient might not correspond to actual connectivity strength, and it is currently unclear whether the observed inter‐individual differences are due to differences between high and moderate correlations or between moderate and low correlations. To address this, it would be beneficial to incorporate monotonic nonlinear transformation functions to adjust the frequency distribution of FC values, maintaining their rank order while allowing for the expansion or compression of differences across specific intervals. Considering the consistency of subsequent algorithm applications, these transformation functions *f* must meet the following criteria:

(2) $$s.t.\left\{\begin{array}{c} \mathrm{dom}\left(f\right)=\mathrm{ran}\left(f\right)=\left[0,1\right]\subset R\\ f\left(x_{1}\right)<f\left(x_{2}\right),\forall x_{1}<x_{2}\\ f\left(0\right)=0\\ f\left(1\right)=1 \end{array}\right.$$

In order to ensure the continuity of the same region in space, a spatial constraint function $\delta_{\mathrm{ij}}$ is also introduced, which retains only the FC values between adjacent voxels in three‐dimensional space and positive values:

(3) $$\delta_{\mathrm{ij}}\left({\mathrm{FC}}_{\mathrm{ij}}\right)=\left\{\begin{array}{c} 1,\text{dist}\left(i,j\right)\leq \sqrt{3}\;\text{and}\;{\mathrm{FC}}_{\mathrm{ij}}>0\\ 0,\text{dist}\left(i,j\right)>\sqrt{3}\;\text{or}\;{\mathrm{FC}}_{\mathrm{ij}}\leq 0 \end{array}\right.$$

where $\text{dist}\left(i,j\right)$ denotes the Euclidean spatial distance between voxel *i* and voxel *j*, with the distance between any two adjacent voxels along each spatial coordinate direction being set to 1. Under the condition of $\text{dist}\left(i,j\right)\leq \sqrt{3}$, the three‐dimensional neighboring voxel set $U_{i}$ of voxel *i* can contain up to 26 voxels. This approach applies constraints on the connections between non‐neighboring voxels that exhibit high similarity (such as the connections of symmetrical voxels in left and right brain regions, etc.), ensuring the spatial continuity of partition after clustering. In this study, the specific meaning of negative correlation values is not considered. When ${\mathrm{FC}}_{\mathrm{ij}}\leq 0$, $\delta_{\mathrm{ij}}\left({\mathrm{FC}}_{\mathrm{ij}}\right)=0$.

Finally, the similarity measurement function is defined as:

(4) $$s_{\mathrm{ij}}=f\left(\delta_{\mathrm{ij}}\left({\mathrm{FC}}_{\mathrm{ij}}\right)\right)$$

Three transform functions are introduced as follows:

(5) $$f_{\text{Root}}\left(x,k\right)=\frac{\left(k+1\right)\sqrt{x}}{1+k\sqrt{x}}$$

(6) $$f_{\text{Square}}\left(x,a\right)=x^{a}$$

(7) $$f_{\text{Gaussian}}\left(x,\sigma \right)=\frac{\exp\left(-\frac{{\left(x-1\right)}^{2}}{2\sigma^{2}}\right)-\exp\left(-\frac{1}{2\sigma^{2}}\right)}{1-\exp\left(-\frac{1}{2\sigma^{2}}\right)}$$

$f_{\text{Root}}$ can widen the difference between low and medium correlation values; $f_{\text{Square}}$ can enlarge the difference between medium and high correlation values. $f_{\text{Gaussian}}$ allows for a smoother distribution of correlation values. For the selection of the hyperparameters of each transformation function, an enumeration method is used in this study to calculate the subsequent clustering performance in order to find a suitable metric construction method. Where, the hyper parameters are set as *k* = 1, *a* = 2 and *σ* = 1, respectively.

Moreover, by introducing the function below:

(8) $$f_{\text{Origin}}\left(x\right)=x$$

and keeping FC's distribution unchanged, it is utilized as a reference for contrasting the effect of different transformation functions on the outcomes.

### 2.4 Grouping Level Clustering Algorithm

Spectral clustering is the fundamental algorithm used for building cerebellar partitioning in this study, implemented through the principle of normalized cut algorithm. The construction of group‐level partitions will employ the idea of cluster ensembles in a two‐level clustering approach.

In the first level of clustering, individual‐level clustering will be performed by defining the similarity matrix among cerebellar voxels of each individual using the above‐mentioned similarity measure. In the second level of clustering, group‐level clustering will be performed by transforming the partition results of all individuals into an adjacency representation matrix and averaging it to form a similarity measure matrix for group‐level clustering.

The definition of the adjacency representation matrix is given as follows. In partition result,

(9) $$C=\left[c_{1},c_{2},\cdots ,c_{n}\right]$$

$c_{i}$ represent the voxel *i*'s partition labels, which range from 1 to *K*. The value of any element $a_{\mathrm{ij}}$ in the adjacency representation matrix AR is defined as:

(10) $$a_{\mathrm{ij}}=\left\{\begin{array}{c} 1,c_{i}=c_{j}\\ 0,c_{i}\neq c_{j} \end{array}\right.$$

This transformation can solve the matching problem of cluster result labels.

Therefore, the similarity measurement matrix used in the second‐level group clustering integrates all individual partition adjacency matrices and is defined as follows:

(11) $$S_{G}=\frac{1}{N}\sum_{n=1}^{N}{\mathrm{AR}}_{n}$$

where *N* is the sample size.

The group‐level partitioning pipeline could be summarized by the pseudocode below (Algorithm 1).

ALGORITHM 1 **Main pipeline of group clustering ensemble.**

**Input:** Functional connectivity matrices set of all subjects $X=\left[{\mathrm{FC}}_{1},{\mathrm{FC}}_{2},\cdots ,{\mathrm{FC}}_{i},\cdots ,{\mathrm{FC}}_{N}\right]$, ${\mathrm{FC}}_{i}$ is a m×m dimensional Functional connectivity matrix, *N* is the number of subjects; number of clusters *k*; transforming function *f*.

**Output:** group partition label array $l_{g}$.

01: **Initializing** $S_{G}=\text{zeros}\left(m,m\right)$

02: **for** *i* = *1*: *N do*

03: $s_{i}=f\left(\delta \left({\mathrm{FC}}_{i}\right)\right)$

04: $l_{i}=\text{SpectralCluster}\left(s_{i},k\right)$

$S_{i}=\text{VecToMat}\left(l_{i}\right)$

$S_{G}=S_{G}+S_{i}$

05: **end for**

06: $S_{G}=S_{G}/N$

07: $l_{G}=\text{SpectralCluster}\left(S_{G},k\right)$

# Convert the partition label array to adjacency representation matrix

**function:** *VecToMat*

**Input:** partition label vector l.

**Output:** adjacency representation matrix *S*.

01: $n=\text{length}\left(l\right)$

**Initializing** $S=\text{zeros}\left(n,n\right)$

**for** *i* = *1*: *n do*

02: $\mathrm{loc}=\text{find}\left(l==l\left(i\right)\right)$

$S\left(i,\mathrm{loc}\right)=1$

$S\left(i,i\right)=0$

03: **end for**

### 2.5 Evaluation Measurements

In the realm of cerebellar segmentation, four key evaluative criteria are delineated as follows: (1) the number of cerebellar partitions is variable and yields similar functional distributions at different numbers of partitions (Bryce et al. 2021); (2) partitioning is reproducible and can yield similar results across different datasets (Arslan et al. 2018; Kurmukov et al. 2020; Nikolaidis et al. 2020); (3) signals within the same partition remain functionally homogeneous (Craddock et al. 2011; Fan et al. 2020); (4) voxels that fall within the same partition are spatially continuous, thereby enhancing the interpretability of the results (Craddock et al. 2011). In this work, the Dice coefficients and Normalized Mutual Information (NMI) values for both group‐level partition and individual partition set were calculated one by one to measure the repeatability of partition. Widely recognized for its utility in numerous studies, the Dice coefficient serves as a principal metric for gauging the similarity between two distinct segmentations. The calculation of this coefficient in the current study is conducted through the following equation:

(12) $$D=\frac{2\left|{\mathrm{AR}}_{X}\times {\mathrm{AR}}_{Y}\right|}{\left|{\mathrm{AR}}_{X}\right|+\left|{\mathrm{AR}}_{Y}\right|}$$

However, with the increase of cluster number, the Dice coefficient tends to drift downward. To address this, the NMI is utilized for supplementary validation, which characterizes the similarity of two kinds of partitions from the perspective of information entropy. The formula for NMI is as follows:

(13) $$\mathrm{NMI}\left(X,Y\right)=\frac{2I\left(X,Y\right)}{H\left(X\right)+H\left(Y\right)}$$

Here,

(14) $$I\left(X,Y\right)=\sum_{x,y}p\left(x,y\right)\log\frac{p\left(x,y\right)}{p\left(x\right)p\left(y\right)}$$

(15) $$H\left(X\right)=-\sum_{x}p\left(x\right)\log p\left(x\right)$$

(16) $$H\left(Y\right)=-\sum_{y}p\left(y\right)\log p\left(y\right)$$

where *X* and *Y* represent the label vectors of the two partitions, respectively. The value range of the two measurement methods is [0, 1]. In this study, higher values are anticipated, indicating a greater degree of similarity and consistency between the partitions.

The signal consistency of the partition was measured by intraregional homogeneity (IRH) and average intraregional homogeneity (AIRH) in this study.

IRH is applied to measure signal consistency within a single partition and is defined as the average Pearson correlation coefficient between all voxel pairs within a partition, providing a measure of the uniformity of signal across the region.

(17) $$\mathrm{IRH}=\frac{2}{\left|C_{k}\right|\left(\left|C_{k}\right|-1\right)}\sum_{x_{i},x_{j}\in C_{k}}\text{Corr}\left(x_{i},x_{j}\right)$$

where $\left|C_{k}\right|$ represents the number of voxels within the *k*‐th partition, $\text{Corr}\left(x_{i},x_{j}\right)$ refers to Pearson correlation, which is calculated in the same way as FC.

AIRH is used to measure the overall signal consistency of the partition, which is defined as the whole brain weighted average of IRH. The proportion of partition voxel numbers to the entire brain is introduced as a weighting factor to avoid extreme effects of unbalanced partitions and to more accurately reflect the comprehensive signal consistency performance of the partition results.

The calculation of AIRH is as follows:

(18) $$\begin{array}{cc} \text{AIRH} & \;=\sum_{k=1}^{K}{\mathrm{IRH}}_{k}=\sum_{k=1}^{K}\frac{\left|C_{k}\right|}{n}\times \frac{2}{\left|C_{k}\right|\left(\left|C_{k}\right|-1\right)}\\ & \;=\frac{2}{n}\sum_{k=1}^{K}\frac{1}{\left(\left|C_{k}\right|-1\right)}\sum_{x_{i},x_{j}\in C_{k}}\text{Corr}\left(x_{i},x_{j}\right) \end{array}$$

where *n* represents the total number of voxels, *K* represents the number of partitions, and other definitions are the same as in the above formula.

### 2.6 Classification Models

This module aims to utilize the aforementioned partitioning scheme to extract cerebellar network features and apply them in PD classification tasks, demonstrating the practical value of the new scheme. First, the mean sequence of BOLD signals within each partition is calculated according to the partition template, and the Pearson correlation of the mean sequences between each partition of the cerebellum is calculated as the FC, and the FC values are normalized as the features in the classification model, and for *N* partitions, *N* (*N* − 1)/2 features can be constructed.

Given the significant age and gender differences between the PD group and the HC group in this study, our approach was to mitigate the impact of these confounding variables by examining how all FC values correlate with age and gender. The Pearson correlation coefficient was used to measure the former, while independent two‐sample *t* tests were employed for the latter. Any FC values associated with age or gender (*p* < 0.05) were excluded. Following normalization, the remaining features were included in a logistic regression model with L2 regularization. To evaluate the classification performance, average accuracy and average area under the receiver operating characteristic curve (AUC) values were calculated using the 5‐fold cross‐validation method.

As the features are standardized, the coefficient of the regression model can reflect its importance. Based on brain regions $X_{1},X_{2},\ldots ,X_{N}$ and paired features ${\mathrm{FC}}_{1,2},{\mathrm{FC}}_{1,3},\cdots ,{\mathrm{FC}}_{N-1,N}$ corresponding coefficients are expressed as $w_{1,2},w_{1,3},\cdots ,w_{N-1,N}$.

The min–max normalization absolute coefficient for $w_{i,j}$ is

(19) $$w_{i,j}^{\prime }=\frac{\left|w_{i,j}\right|}{\max\left\{\left|w_{1,2}\right|,\left|w_{1,3}\right|,\cdots ,\left|w_{N-1,N}\right|\right\}}$$

At this moment, the importance assessment Imp of the brain region $X_{i}$ is defined as the normalized coefficient of its associated FC, which is expressed as follows:

(20) $${\mathrm{Imp}}_{i}=\frac{1}{N-1}\sum_{j\neq i}w_{i,j}^{\prime }$$

Final representation of brain region feature importance is expressed in a normalized form:

(21) $${\bar{\mathrm{Imp}}}_{i}=\frac{{\mathrm{Imp}}_{i}}{\max{\left\{{\mathrm{Imp}}_{i}\right\}}_{i=1,2,\cdots ,N}}$$

The larger the numerical value, the more important it is, and the highest value for the brain region coefficient is 1.

## 3 Results

### 3.1 Reproducibility Study Employing Diverse Similarity Metrics

**FIGURE 2:**
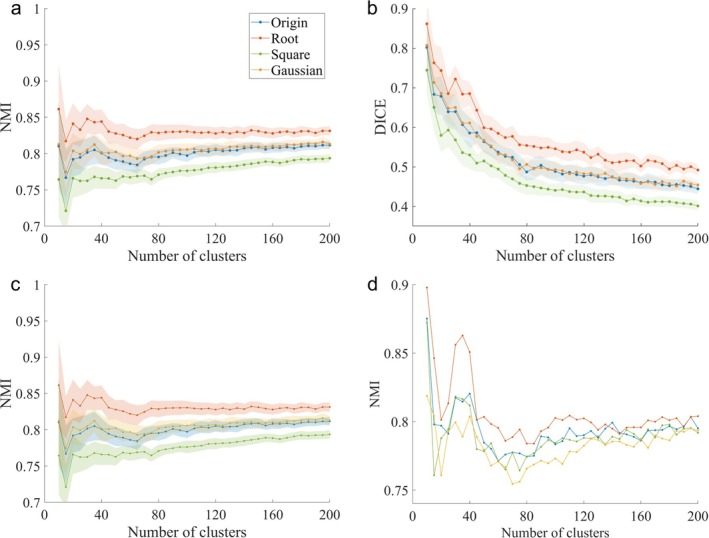
Comparison of partition repeatability under different transformations. (a, b) The NMI similarity and Dice coefficient similarity under the HCP‐based data, respectively. (c) The NMI similarity under the Southwest Hospital dataset. (d) The NMI similarity between partitions based on the HCP dataset and partitions under the Southwest Hospital dataset.

We employed the NMI and Dice coefficients to assess the reproducibility of the partitions, with higher values indicating a higher degree of reproducibility. Figure 2 illustrates the relationship between the number of partitions and reproducibility under various similarity metrics. In Figure 2a,b, we present the NMI and Dice coefficient between the group‐level partition and the individual partition of the validation set, established based on the HCP dataset and involving different transformations, respectively (Figure S1 displays the changes in the FC distribution due to the four transformation functions). Figure 2c portrays the NMI results for cerebellar partitions under distinct similarity transformations, based on the Southwest Hospital dataset, with trends that generally align with the NMI values obtained from the HCP data. Figure 2d highlights the variations in NMI between partitions constructed from the Southwest Hospital dataset and those from the HCP dataset.

Table 2 details NMI differences for select partition numbers. Our findings indicate that the Root series of partitions exhibits greater partition similarity (Root > Gaussian ≈ Origin > Square; *F* value = 194.08, *p* value < 10^−3^, ANOVA). The NMI similarity progressively increases as the number of partitions rises, while the Dice coefficient displays a pronounced decline. Figure S2 showcases a selection of representative cerebellar partitions from the Root series, further illustrating the nuances of partition similarity under diverse conditions.

**TABLE 2:** NMI of different similarity measurements.

Number of clustering	10	30	50	100	150	200
Origin	0.8249	0.7986	0.7935	0.7904	0.7957	0.7994
Root^a^	0.8686^a^	0.8436^a^	0.8112^a^	0.8133^a^	0.8156^a^	0.8172^a^
Square	0.7836	0.7653	0.7704	0.7698	0.7774	0.7817
Gaussian	0.8287	0.8068	0.7955	0.7919	0.7978	0.8032

^a^ The best similarity measurement and its NMIs.

### 3.2 Signal Homogeneity Analysis

To explore the signal homogeneity within the partitions, a newly generated suite of partitions based on the Spatially Unbiased Infratentorial Template (SUIT) was used, the Structural Functional Fusion Model of the Cerebellum formulated by Pezoulas, the Multi‐Domain Task Set Template (MDTB) derived from Task Experiment datasets, and the Cerebellar17 Network developed by Buckner. In assessing signal homogeneity, the Intraregional Homogeneity (IRH) was employed to compare localized subregions' signal consistency. Furthermore, the Average Intraregional Homogeneity (AIRH), constructed through a weighted average of IRH values, was employed to evaluate the overall signal homogeneity across the sub‐areas.

**FIGURE 3:**
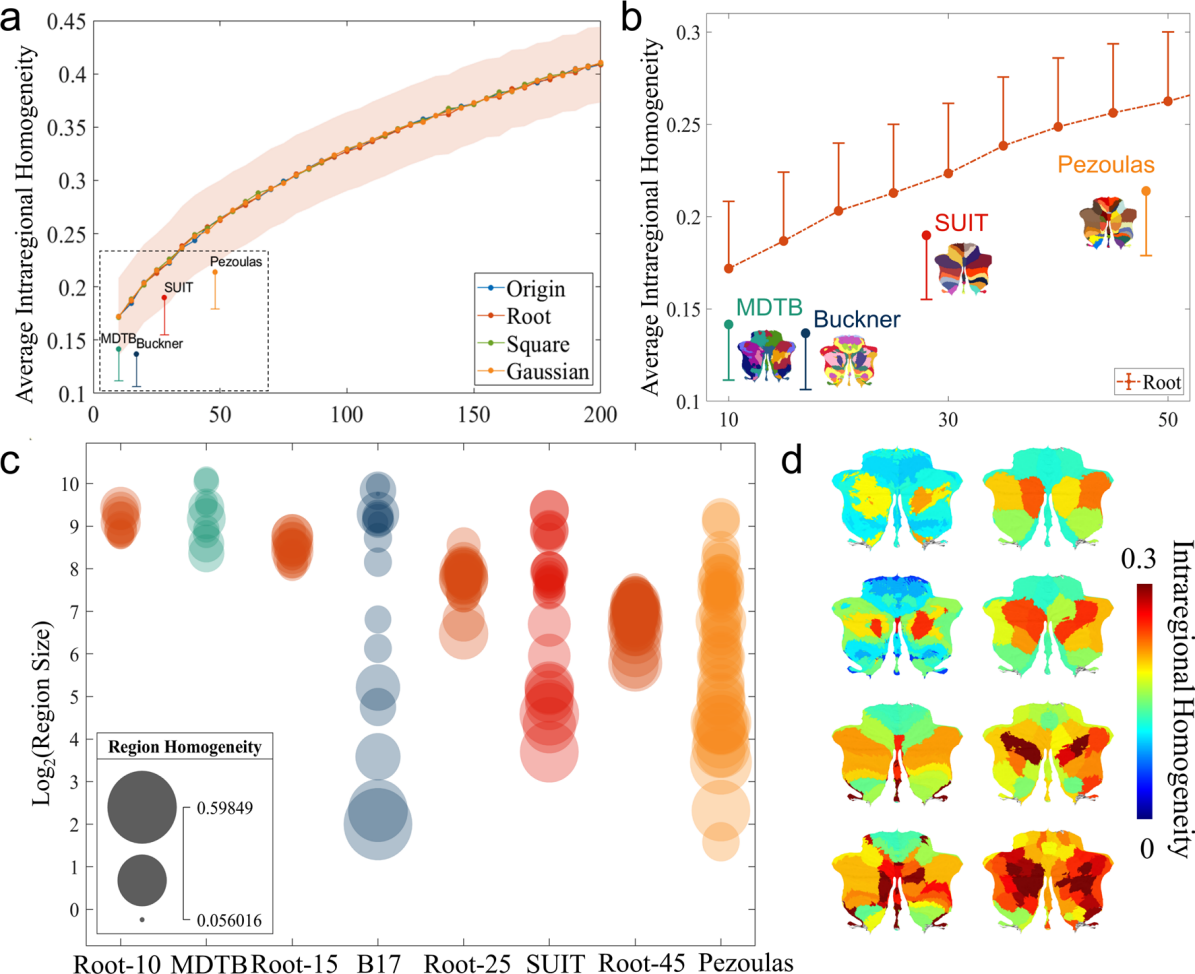
Comparison of homogeneity of newly constructed series of partitions with open partitions. (a) The AIRH of the four new partitions versus the AIRH of the four open partitions; (b) illustrated in detail the AIRH of the Root series of partitions versus the AIRH of the four public partitions; (c) the IRH bubble plots for the eight partitions, where the bubble size is positively correlated with the IRH value, and the bubble height represents the log2 (area size) value; (d) indicates the visualization of IRH intensity in the cerebellar plane for the eight subdivisions (threshold: 0.3).

Figure 3a illustrates the AIRH results for the newly generated series of partitions, revealing an increase in AIRH as the number of partitions escalates. Importantly, the intra‐regional homogeneity remains consistent across all four transformation functions. In Figure 3b, we provided a detailed AIRH comparison between the four open templates and the Root series of partitions for clusters ranging from 10 to 50. A comprehensive analysis involving a number of Root partitions similar to that of the open template partitions unveils superior regional homogeneity in the Root series partitions. Table 3 encapsulates the results of the Root series partition comparisons with the open templates when the partition numbers closely align. All comparisons yield statistically significant differences (*p* value < 10^−11^), with the most substantial disparity observed between Root45 and the Pezoulas partitioning template (*t* value = 23.401, *p* value < 10^−18^, paired *t*‐test), while the most modest discrepancy is evident between Root25 and the SUIT partition template (*t* value = 12.624, *p* value < 10^−11^, paired *t*‐test).

**TABLE 3:** Comparison of global homogeneity AIRH across different partitions.

Root series	AIRH	Open templates	AIRH	*t* ^a^	*p* ^b^
Root10	0.1719 ± 0.0364	MDTB	0.1416 ± 0.0301	16.5732	2.4429 × 10^−15^
Root15	0.1869 ± 0.0373	Buckner	0.1369 ± 0.0305	16.4638	2.8603 × 10^−15^
Root25	0.2128 ± 0.0372	SUIT	0.1899 ± 0.0347	12.6240	1.3516 × 10^−12^
Root45	0.2562 ± 0.0374	Pezoulas	0.2139 ± 0.0349	23.4010	5.3987 × 10^−19^

^a^ *t* values calculated using paired *t*‐tests, respectively (Root Series—Open Template).

^b^ *p* values calculated using paired *t*‐tests, respectively (Root Series—Open Template).

Figure 3c presents a series of comparative bubble plots that illustrate the IRH between the Root series partitions and other open partitions, where the number of clustered partitions is 10, 15, 25, and 45, respectively. The results vividly demonstrate that the Root series of partitions consistently exhibits higher IRH values when compared to other open partitions featuring subregions of equivalent size. Moving on to Figure 3d, this figure provides a planar visualization of the cerebellum for each division of IRH as initially depicted in Figure 3a. The Root series of partitions consistently showcases superior homogeneity, particularly in the medial cerebellar Crus region and posterior lobe region. Furthermore, the cerebellar planimetry among the open partitions reveals a recurring pattern, indicating that the medial cerebellar region often exhibits stronger IRH values in comparison to the lateral region.

### 3.3 Cerebellar Partition Spatial Character Analysis

**FIGURE 4:**
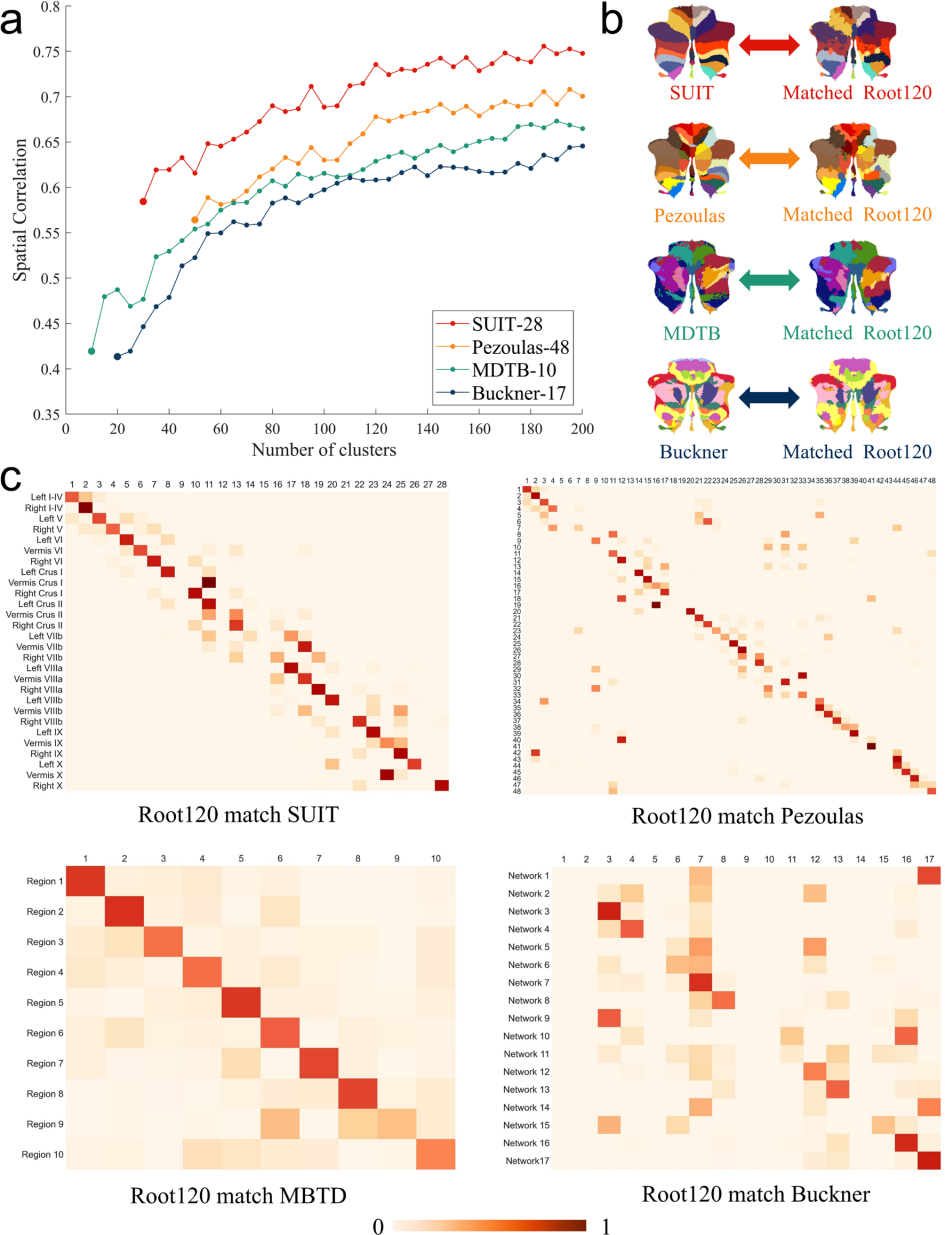
Spatial similarity comparison. (a) The spatial similarity between the Root partition and the open partition; (b) the open partition templates and the matching partition template for the Root120 counterpart, parts with consistent colors represent areas where the open template matches the Root120 partition; (c) the region‐related heat maps after matching the Root120 with the open partition.

To measure their spatial correlation, we employed the Dice coefficient, a reliable indicator of template similarity. Figure 4a portrays the Dice spatial correlation values between the Root series partitions and the four open templates across varying numbers of clusters. With an escalating number of clusters, the Root series partitions exhibit increasing refinement and higher spatial similarity to the other templates. Notably, the Root series partitions demonstrate the strongest resemblance to the SUIT template. According to the results in Figure 4a, the spatial similarity between the Root series partition and each of the open templates tends to be stabilized when the number of partitions reaches 120, so the representative Root120 partition is selected for the subsequent matching study. Figure 4b shows the brain region visualization results after the Root120 partition was matched with the four open templates labeled according to the winner‐take‐all principle. The results show that the label‐matched Root120 partitions have similar spatial distribution patterns to all four open templates. The heat map in Figure 4c illustrates the spatial alignment of the Root120 partition with the SUIT, Pezoulas, Buckner, and MDTB templates. The results show that the Root120 cerebellar partition has a superior correct match on the main diagonal with the SUIT and Pezoulas partitions.

### 3.4 Classification Performance Analysis

**FIGURE 5:**
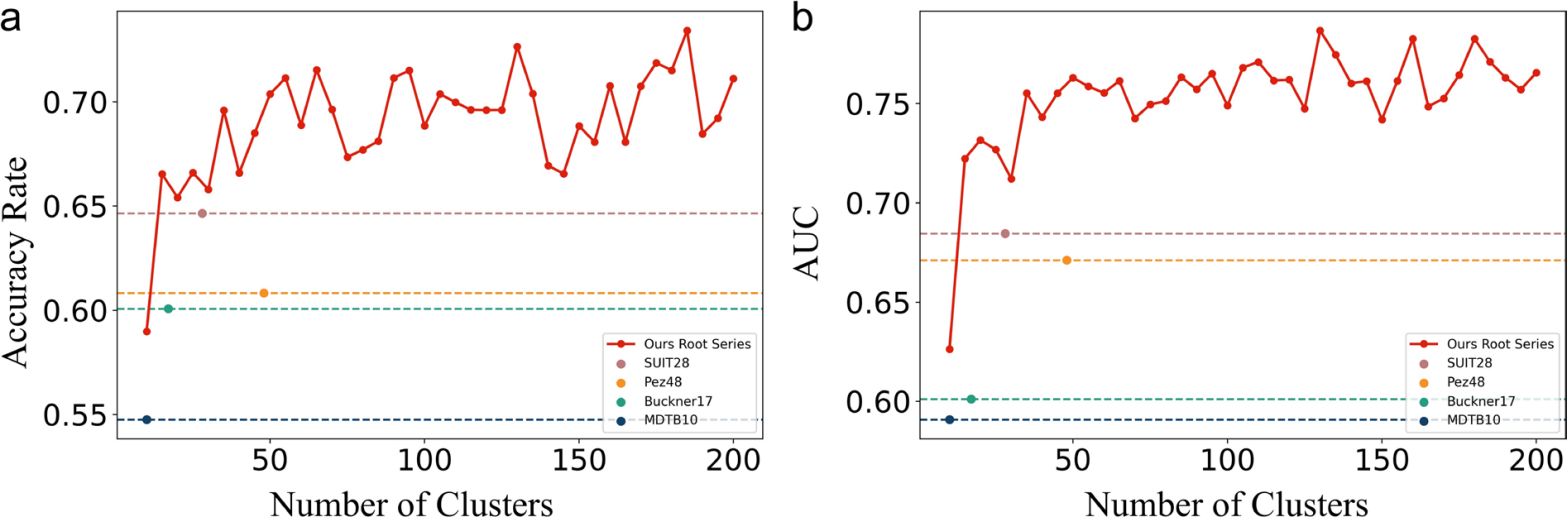
Effectiveness of FC feature classification model based on extraction from different cerebellar partitions. (a) Average accuracy rate; (b) average AUC.

In the classification model, normalized FC in individual brain regions from the root series partition as well as from four different open templates were used as features. Subsequently, these features underwent normalization and were integrated into the L2 regularized logistic regression classification algorithm. The performance of each classification model was assessed through the accuracy rate and the AUC value, employing a 5‐fold cross‐validation methodology. The detailed information is shown in Figure 5, Tables 4 and 5.

**TABLE 4:** Classification accuracy and AUC values under FC features extracted based on open templates.

Open template	Accuracy	AUC
MDTB	0.5476 ± 0.0234	0.5909 ± 0.0598
Buckner	0.6008 ± 0.0826	0.6012 ± 0.1136
SUIT^a^	0.6466 ± 0.0604^a^	0.6846 ± 0.0796^a^
Pezoulas	0.6083 ± 0.0810	0.6712 ± 0.1018

^a^ Optimal performance results.

**TABLE 5:** Classification accuracy and AUC values under FC features extracted based on Root's cerebellar partitioning.

Root series partition	Accuracy	AUC
Root10	0.5898 ± 0.1246	0.6264 ± 0.1184
Root15	0.6653 ± 0.0769	0.7224 ± 0.0940
Root25	0.6660 ± 0.1059	0.7268 ± 0.1272
Root45	0.6850 ± 0.0991	0.7553 ± 0.1009
Root65	0.7154 ± 0.0786	0.7615 ± 0.0903
Root130^a^	0.7265 ± 0.0605	0.7868 ± 0.0657^a^
Root160	0.7076 ± 0.0748	0.7827 ± 0.0796
Root180	0.7151 ± 0.0536	0.7827 ± 0.0571
Root185^a^	0.7342 ± 0.0477^a^	0.7711 ± 0.0671

^a^ Optimal performance results.

Figure 5a illustrates the average accuracy of FC feature classification models derived from various cerebellar partition extractions. Within the feature model based on Root partition extraction, the average classification accuracy displays a gradual increase as the number of partitions rises, following an initial ascent. Notably, the lowest classification accuracy is observed at a partition number of 10 (average classification accuracy: 0.590), with the optimal performance achieved at a partition number of 185 (average classification accuracy: 0.734). Comparatively, among the feature models extracted from open partitions, the lowest‐performing features are those derived from the MDTB partitions (average classification accuracy: 0.548). Conversely, the SUIT partition extraction delivers the most robust performance (average classification accuracy: 0.647). Consistently, the classification model based on Root partitions consistently outperforms those constructed using open partitions, enhancing the average classification accuracy by 0.087 at its optimal partition number.

Figure 5b illustrates the AUC of FC feature classification models, which parallels the pattern trend observed in Figure 5a. Table 4 presents the accuracy, average AUC, and standard deviation of four open templates, with their optimal results clearly indicated. Meanwhile, Table 5 provides analogous metrics for eight representative Root partitions, highlighting their respective optimal results. In the feature model extracted from Root partitions, the lowest AUC value is observed at a partition number of 10 (average AUC: 0.626), with optimal performance achieved at a partition number of 130 (average AUC: 0.787). Among the feature models extracted from open partitions, the MDTB partitions exhibit the weakest performance (average AUC: 0.591), while the most robust performance is noted in the feature model extracted from SUIT partitions (average AUC: 0.685). Notably, the classification model utilizing Root partitioning consistently outperforms those constructed based on open partitions, enhancing the average AUC metric by 0.103 at its optimal partition number.

To gauge the influence of cerebellar partitions on the classification model, we determined the feature importance of brain partitions. The significance of FC features was assessed using the results from 5‐fold cross‐validation across a variety of models. Subsequently, the FC features were correlated with their respective brain regions, thus generating the feature importance scores for these areas.

**FIGURE 6:**
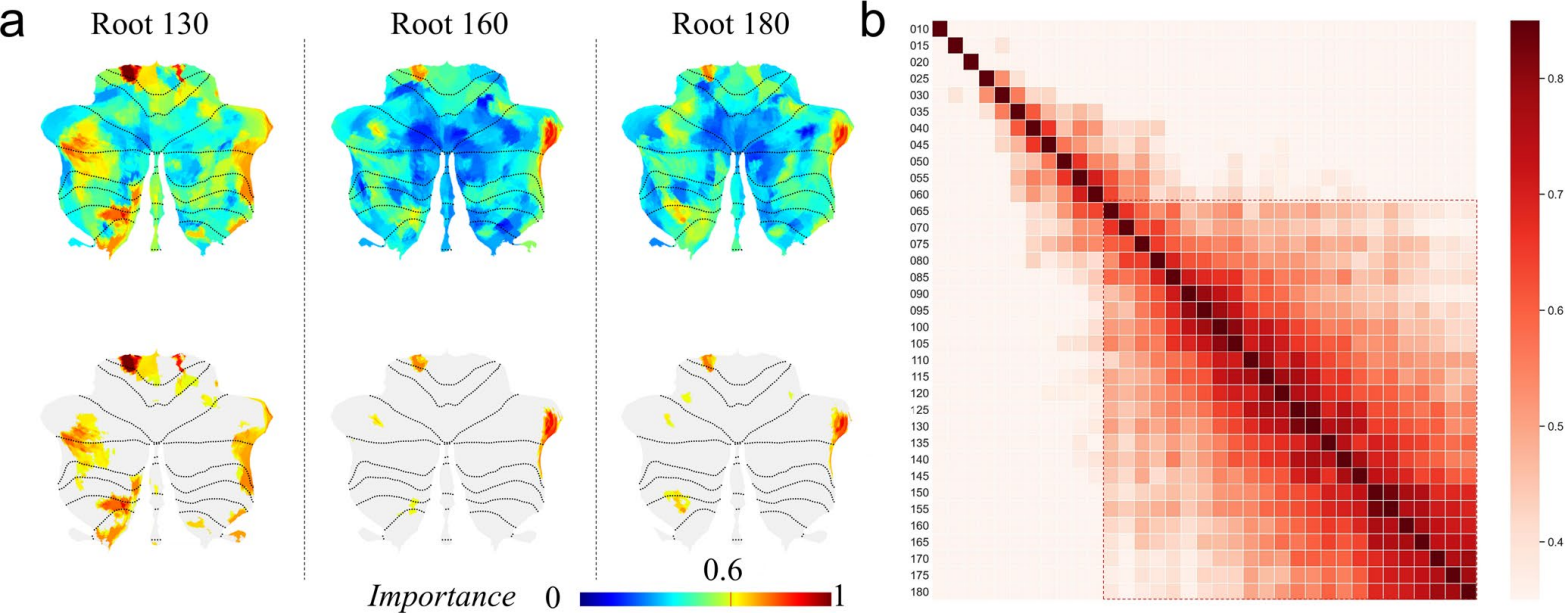
Results of feature importance of brain regions based on classification models with different partitions. (a) The top plot shows the normalized feature importance planes for Root130, Root160, and Root180, and the bottom plot represents the screening results after threshold correction (Imp > 0.6); (b) shows heat maps related to the importance of features in different partitions (*r* > 0.35, *p* < 10^−172^; *r* > 0.45, *p* < 10^−303^).

Based on the results of the previous classification model, we selected three cerebellar partitions (Root130, Root160, and Root180) with better classification and more stable performance as representative results of the spatial distribution of cerebellum for feature importance. Figure 6a showcases selected partitions (Root130, Root160, and Root180) as representatives of feature region importance results, highlighting their superior performance in the classification model. All top three plots in Figure 6a underscore the substantial feature importance of the left cerebellum, the right cerebellar limbic region, and the anterior cerebellar lobe region, whereas the medial regions of Crus I and Crus II in lobule VI exhibit comparatively weaker feature importance. The bottom plot in Figure 6a provides a visualization of the crucial features filtered for the top plot (threshold: 0.6). This visualization reveals that in the Root130 partition, important features are predominantly distributed in the bilateral anterior cerebellar lobes, the left posterior cerebellar lobe, and the bilateral cerebellar crus regions. In the Root160 partition, the critical features are primarily located in the left posterior cerebellar lobe, the left anterior cerebellar lobe, and the right cerebellar limbic regions. In the Root180 partition, the significant features are mainly concentrated in the posterior left cerebellar lobe, the left anterior cerebellar lobe, and the right cerebellar marginal region. Figure 6b presents a heat map illustrating the correlation of feature importance results across different partitions in the classification models. Each color block in the heat map denotes the similarity of feature importance between the corresponding two partitions. The diagonal section of the heat map reveals a strong concordance in feature importance results for each partition. Moreover, the homogeneity between partitions becomes more pronounced as the partitions become more finely detailed.

## 4 Discussion

The functions of the human cerebellum are likely to exhibit similar diversity and complexity to those of the cerebral cortex. (Ren et al. 2019). Not only that, but different brain atlases need to be specified for different research questions (e.g., different neuropsychiatric disorders), and the cerebellum is more individual‐specific than the cortex (Li et al. 2023; Marek et al. 2018), so that a detailed functional partition of the cerebellum which helps us elucidate this diverse functionality and connectivity is essential. In our study, we proposed a novel cerebellar partitioning method designed to offer a variable number of partitions, to enhance partition reproducibility, and to ensure both regional homogeneity and spatial continuity. We applied the approach to the two independent datasets to validate the effectiveness of this partitioning series. Subsequently, we used this method for a PD classification problem as a clinical practice.

### 4.1 Reproducibility of the Novel Cerebellar Partitions

We calculated the NMI and the Dice coefficient to measure the similarity between individual partitions of the validation set and group‐level partitions derived from the training set under various transformations. Unlike other cerebellar open partitions, which have unique construction processes and are thus non‐reproducible, we retained the original FC matrix as a similarity matrix in this study. This served as a reference outcome based on the conventional approach. The results of the reproducibility study for both the HCP dataset and the Southwest Hospital dataset demonstrated the stability of our cerebellar partitioning method. Notably, employing the Root transformation function significantly enhances the reproducibility of the partitions, whereas the Square transformation function significantly diminishes the reproducibility of the partitions. These functions represent order‐preserving mappings, indicating that the FC consistently characterizes voxel correlations. However, the reliability of FC metrics has been debated (Andellini et al. 2015; Varikuti et al. 2016; Zuo and Xing 2014), and prior studies have often sought to enhance the reliability of results through new algorithms (Cheng and Liu 2021; Wang et al. 2016). In contrast, our study commences from the original metrics, hypothesizing that altering the FC distribution may influence the reliability of results. Our experiments confirmed that expanding the FC distribution toward values close to 1 substantially improves the reliability of the partitioning results, whereas compressing the FC distribution toward values close to 0 leads to decreased reliability. This effect may be attributed to the Root transformation expanding the disparity between high and low correlation values, making it easier to discern cut edges between boundary points. Conversely, the square transformation compresses this disparity, facilitating the recognition of correlations between boundary points and reducing their correlation with other homoplastic elements, leading to lower reproducibility. Furthermore, the group‐level construction method based on ensemble clustering also contributes to enhanced partitioning reproducibility (Kurmukov et al. 2020; Nikolaidis et al. 2020). However, it is important to note that this aspect of our method was not rigorously validated in this study.

### 4.2 Regional Signal Homogeneity and Spatial Continuity of Newly Constructed Cerebellar Partitions

The newly constructed partitions in our approach benefit from appropriate spatial structure constraints and the stability of the spectral clustering algorithm when applied to brain image data. These factors result in partitions that exhibit pronounced spatial continuity and regional signal homogeneity, aligning with prior research (Arslan et al. 2018; Craddock et al. 2011; Dillon and Wang 2020). The incorporation of spatial adjacency as a constraint in the construction of similarity metrics ensures that voxels within the same subregion are interconnected, thereby contributing to the preservation of spatial continuity in the newly constructed partitions. Additionally, the application of the Normalized Cuts algorithm aids in maintaining partition sizes that are relatively uniform (Bajada et al. 2020). As the partitions become more refined, there is an observed increase in the signal homogeneity within these partitions. An examination of existing open templates reveals that MDTB partitions and Buckner17 network partitions tend to exhibit lower signal homogeneity, whereas SUIT partitions and Pezoulas partitions display stronger signal homogeneity. This distinction arises from the spatial discontinuity in the former two partitions (Buckner et al. 2011; King et al. 2019) and the spatial continuity in the latter two partitions (Pezoulas et al. 2019; Rolls et al. 2020). These findings support the notion that spatial continuity contributes to achieving the signal homogeneity of partitions. Further comparisons of signal consistency among Root10, Root15, MDTB, and Buckner partitions indicated that partitions with more uniform sizes are more likely to demonstrate stronger signal homogeneity. Although the Buckner17 network includes a greater number of partitions than the MDTB, the individual region sizes are more heterogeneous, which may explain the lower signal coherence in Buckner17 as compared to MDTB partitions.

### 4.3 Number of Partitions for Newly Constructed Cerebellar Partitions

One notable feature of the cerebellar partitions constructed in this study is their adaptability in the number of partitions. Nevertheless, it is imperative to validate the consistency of the partitioning outcomes across various partition counts, aligning with existing literature (Bryce et al. 2021; Zhi et al. 2022). To assess this stability, we calculated the spatial correlation between the Root partition and other templates at varying partition numbers using the winner‐take‐all algorithm, with the four open templates serving as the reference standard (Buckner et al. 2011; Xue et al. 2021). Our results revealed that the spatial correlation with each template increases as the number of partitions rises, indicating that the stability of the partition results improves with an increasing number of partitions. Furthermore, we observed that the Root series partitions exhibit higher spatial correlation with structurally bounded partitions, such as SUIT partitions and Pezoulas partitions. This suggested that the Root series partitions are adept at capturing the cerebellar structural information. This enhanced correlation may be attributed to the more stable organizational boundaries and the greater variability in functional data (Cassidy et al. 2018; Griffanti et al. 2016; Telesford et al. 2013). By integrating the individual partitions of the validation set through the cluster ensemble technique, the heterogeneity of stable organizational boundary voxels becomes more discernible, resulting in stronger similarity with the structurally constrained partitions. The optimal number of partitions is not explicitly given in this study. From the results of the evaluation indexes, in the evaluation of partition repeatability, the optimum is reached when the number of partitions is 10, and the local optimum is reached when the number of partitions is 30; in the evaluation of signal homogeneity, the optimum signal homogeneity is reached when the number of partitions is 200; and the optimum classification accuracy is reached when the number of partitions is 185 in the classification model of PD. This shows that the determination of the “optimal partition” should not be arbitrarily based on a certain index but should be based on the needs of the actual study to select the appropriate coarse‐grained partition (Craddock et al. 2011; Ren et al. 2019; Seghier 2018). In subsequent studies, the use of different fine‐grained partitions to test the stability of the network analysis results is also a more appropriate way of retesting, which can help researchers to improve the reliability of the results of the cerebellar study.

### 4.4 Application of Newly Constructed Cerebellar Partitioning in PD Classification Modeling

The results of the classification model based on the Root series of partitions and the four open partitions (MDTB, Buckner, SUIT, and Pezoulas) of the normalized FC in a 5‐fold cross‐validation setup show that the model using the Root130 and Root180 partitions performs well. The AUC for the Root130 partition model reached 0.787, and the accuracy for the Root185 partition model reached 0.734. These results marked a notable improvement of 0.103 in the AUC evaluation and 0.087 in classification accuracy over the optimal publicly partitioned feature model. Feature importance analysis under different partitions revealed that at a high number of partitions, the classification performance tends to plateau, suggesting that increasing the partition count beyond a certain point does not yield further improvements in model accuracy. Once the partition count surpassed 60, the feature importance for each model exhibited a high degree of correlation, indicating that the results had reached a stable state. Feature importance analysis for the regions using Root130, Root160, and Root180 as representative partitions highlighted that the crucial features were predominantly situated in the anterior cerebellar lobe, the left posterior cerebellar lobe, and the right limbic region of the cerebellum. The anterior cerebellar lobe and the left posterior cerebellar lobe are known to be implicated in motor tasks, such as movement and motor control (Schmahmann et al. 2019), while the right limbic region of the cerebellum is linked to cognitive functions, such as attention and distraction (King et al. 2019). These regions are often affected in Parkinson's disease (Huang et al. 2022; Li et al. 2023), and their characteristic motor and control deficits may be related to the prominently featured brain regions identified in this study. These insights can guide the selection of cerebellar features in the development of future diagnostic models for PD.

## 5 Conclusion

In this study, we introduced a novel approach to cerebellar functional partitioning that integrates spatial information with order‐preserving nonlinear transformations. The partitioning method not only had good spatial continuity as well as partitioning variability, but also verified the high signal homogeneity and stable reproducibility across different datasets through its application to cerebellar functional partitioning. Given that the requirements for brain network node size and complexity may vary across studies, our newly proposed method offered the flexibility to adjust the number of partitions, thereby altering the brain network structure to suit diverse scientific inquiries. Moreover, the features derived from these novel cerebellar partitions had shown promising results in the classification of PD. This suggested that, moving forward, there is potential to further explore biomarkers for a range of diseases leveraging this cerebellar partitioning strategy. The adaptability of our method to cater to the varying demands of brain network analysis is a significant advantage, allowing researchers to tailor the partitioning process to align with the specific objectives of their studies. Furthermore, the successful application of these partitions in disease classification opens up new avenues for biomarker discovery, which could ultimately contribute to more personalized and effective diagnostic and therapeutic approaches.

## Supporting information


**Data S1.** Supporting Information.

## Data Availability

Research data are not shared.

## References

[hbm70268-bib-0001] Amico, E. , and J. Goñi . 2018. “The Quest for Identifiability in Human Functional Connectomes.” Scientific Reports 8, no. 1: 8254. 29844466 10.1038/s41598-018-25089-1 PMC5973945

[hbm70268-bib-0002] Andellini, M. , V. Cannatà , S. Gazzellini , B. Bernardi , and A. Napolitano . 2015. “Test‐Retest Reliability of Graph Metrics of Resting State MRI Functional Brain Networks: A Review.” Journal of Neuroscience Methods 253: 183–192. 10.1016/j.jneumeth.2015.05.020. 26072249

[hbm70268-bib-0003] Arslan, S. , S. I. Ktena , A. Makropoulos , E. C. Robinson , D. Rueckert , and S. Parisot . 2018. “Human Brain Mapping: A Systematic Comparison of Parcellation Methods for the Human Cerebral Cortex.” NeuroImage 170: 5–30. 10.1016/j.neuroimage.2017.04.014. 28412442

[hbm70268-bib-0004] Bajada, C. J. , L. Q. Costa Campos , S. Caspers , et al. 2020. “A Tutorial and Tool for Exploring Feature Similarity Gradients With MRI Data.” NeuroImage 221: 1–11. 10.1016/j.neuroimage.2020.117140. PMC7116330 32650053

[hbm70268-bib-0005] Bryce, N. V. , J. C. Flournoy , J. F. Guassi Moreira , et al. 2021. “Brain Parcellation Selection: An Overlooked Decision Point With Meaningful Effects on Individual Differences in Resting‐State Functional Connectivity.” NeuroImage 243: 118487. 10.1016/j.neuroimage.2021.118487. 34419594 PMC8629133

[hbm70268-bib-0006] Buckner, R. L. , F. M. Krienen , A. Castellanos , J. C. Diaz , and B. T. T. Yeo . 2011. “The Organization of the Human Cerebellum Estimated by Intrinsic Functional Connectivity.” Journal of Neurophysiology 106, no. 5: 2322–2345. 10.1152/jn.00339.2011. 21795627 PMC3214121

[hbm70268-bib-0007] Cassidy, B. , F. D. Bowman , C. Rae , and V. Solo . 2018. “On the Reliability of Individual Brain Activity Networks.” IEEE Transactions on Medical Imaging 37, no. 2: 649–662. 10.1109/tmi.2017.2774364. 29408792

[hbm70268-bib-0008] Cheng, H. , and J. Liu . 2021. “Concurrent Brain Parcellation and Connectivity Estimation via Co‐Clustering of Resting State fMRI Data: A Novel Approach.” Human Brain Mapping 42, no. 8: 2477–2489. 10.1002/hbm.25381. 33615651 PMC8090776

[hbm70268-bib-0009] Craddock, R. C. , G. A. James , P. E. Holtzheimer , X. P. Hu , and H. S. Mayberg . 2011. “A Whole Brain fMRI Atlas Generated via Spatially Constrained Spectral Clustering.” Human Brain Mapping 33, no. 8: 1914–1928. 10.1002/hbm.21333. 21769991 PMC3838923

[hbm70268-bib-0010] Diedrichsen, J. , and E. Zotow . 2015. “Surface‐Based Display of Volume‐Averaged Cerebellar Imaging Data.” PLoS One 10, no. 7: e0133402. 10.1371/journal.pone.0133402. 26230510 PMC4521932

[hbm70268-bib-0011] Dillon, K. , and Y.‐P. Wang . 2020. “Resolution‐Based Spectral Clustering for Brain Parcellation Using Functional MRI.” Journal of Neuroscience Methods 335: 108628. 10.1016/j.jneumeth.2020.108628. 32035090 PMC7061089

[hbm70268-bib-0012] Eickhoff, S. B. , B. T. T. Yeo , and S. Genon . 2018. “Imaging‐Based Parcellations of the Human Brain.” Nature Reviews Neuroscience 19, no. 11: 672–686. 10.1038/s41583-018-0071-7. 30305712

[hbm70268-bib-0013] Fan, L. , Q. Zhong , J. Qin , et al. 2020. “Brain Parcellation Driven by Dynamic Functional Connectivity Better Capture Intrinsic Network Dynamics.” Human Brain Mapping 42, no. 5: 1416–1433. 10.1002/hbm.25303. 33283954 PMC7927310

[hbm70268-bib-0014] Glasser, M. F. , S. N. Sotiropoulos , J. A. Wilson , et al. 2013. “The Minimal Preprocessing Pipelines for the Human Connectome Project.” NeuroImage 80: 105–124. 10.1016/j.neuroimage.2013.04.127. 23668970 PMC3720813

[hbm70268-bib-0015] Gordon, E. M. , T. O. Laumann , B. Adeyemo , J. F. Huckins , W. M. Kelley , and S. E. Petersen . 2016. “Generation and Evaluation of a Cortical Area Parcellation From Resting‐State Correlations.” Cerebral Cortex 26, no. 1: 288–303. 10.1093/cercor/bhu239. 25316338 PMC4677978

[hbm70268-bib-0016] Griffanti, L. , M. Rolinski , K. Szewczyk‐Krolikowski , et al. 2016. “Challenges in the Reproducibility of Clinical Studies With Resting State fMRI: An Example in Early Parkinson's Disease.” NeuroImage 124, no. Pt A: 704–713. 10.1016/j.neuroimage.2015.09.021. 26386348 PMC4655939

[hbm70268-bib-0017] Huang, Z. , H. Lei , G. Chen , et al. 2022. “Parkinson's Disease Classification and Clinical Score Regression via United Embedding and Sparse Learning From Longitudinal Data.” IEEE Transactions on Neural Networks and Learning Systems 33, no. 8: 3357–3371. 10.1109/tnnls.2021.3052652. 33534713

[hbm70268-bib-0018] Ji, J. L. , M. Spronk , K. Kulkarni , G. Repovš , A. Anticevic , and M. W. Cole . 2019. “Mapping the Human Brain's Cortical‐Subcortical Functional Network Organization.” NeuroImage 185: 35–57. 10.1016/j.neuroimage.2018.10.006. 30291974 PMC6289683

[hbm70268-bib-0019] King, M. , C. R. Hernandez‐Castillo , R. A. Poldrack , R. B. Ivry , and J. Diedrichsen . 2019. “Functional Boundaries in the Human Cerebellum Revealed by a Multi‐Domain Task Battery.” Nature Neuroscience 22, no. 8: 1371–1378. 10.1038/s41593-019-0436-x. 31285616 PMC8312478

[hbm70268-bib-0020] Kurmukov, A. , A. Mussabaeva , Y. Denisova , et al. 2020. “Optimizing Connectivity‐Driven Brain Parcellation Using Ensemble Clustering.” Brain Connectivity 10, no. 4: 183–194. 10.1089/brain.2019.0722. 32264696 PMC7247040

[hbm70268-bib-0021] Li, Y. , A. Liu , L. Li , et al. 2023. “Connectivity‐Based Brain Parcellation for Parkinson's Disease.” IEEE Transactions on Biomedical Engineering 70, no. 5: 1539–1552. 10.1109/tbme.2022.3222072. 36378799

[hbm70268-bib-0022] Liu, M. , H. Zhang , F. Shi , and D. Shen . 2023. “Hierarchical Graph Convolutional Network Built by Multiscale Atlases for Brain Disorder Diagnosis Using Functional Connectivity.” IEEE Transactions on Neural Networks and Learning Systems 35, no. 11: 15182–15194. 10.1109/tnnls.2023.3282961. 37339027

[hbm70268-bib-0023] Marek, S. , J. S. Siegel , E. M. Gordon , et al. 2018. “Spatial and Temporal Organization of the Individual Human Cerebellum.” Neuron 100, no. 4: 977–993e977. 10.1016/j.neuron.2018.10.010. 30473014 PMC6351081

[hbm70268-bib-0024] Nettekoven, C. , D. Zhi , L. Shahshahani , et al. 2024. “A Hierarchical Atlas of the Human Cerebellum for Functional Precision Mapping.” Nature Communications 15, no. 1: 8376. 10.1038/s41467-024-52371-w PMC11436828 39333089

[hbm70268-bib-0025] Nikolaidis, A. , A. Solon Heinsfeld , T. Xu , P. Bellec , J. Vogelstein , and M. Milham . 2020. “Bagging Improves Reproducibility of Functional Parcellation of the Human Brain.” NeuroImage 214: 116678. 10.1016/j.neuroimage.2020.116678. 32119986 PMC7302537

[hbm70268-bib-0026] Pezoulas, V. C. , K. Michalopoulos , M. A. Klados , S. Micheloyannis , N. G. Bourbakis , and M. Zervakis . 2019. “Functional Connectivity Analysis of Cerebellum Using Spatially Constrained Spectral Clustering.” IEEE Journal of Biomedical and Health Informatics 23, no. 4: 1710–1719. 10.1109/jbhi.2018.2868918. 30188842

[hbm70268-bib-0027] Ren, Y. , L. Guo , and C. C. Guo . 2019. “A Connectivity‐Based Parcellation Improved Functional Representation of the Human Cerebellum.” Scientific Reports 9, no. 1: 9115. 10.1038/s41598-019-45670-6. 31235754 PMC6591283

[hbm70268-bib-0028] Rolls, E. T. , C. C. Huang , C. P. Lin , J. Feng , and M. Joliot . 2020. “Automated Anatomical Labelling Atlas 3.” NeuroImage 206: 116189. 10.1016/j.neuroimage.2019.116189. 31521825

[hbm70268-bib-0029] Schaefer, A. , R. Kong , E. M. Gordon , et al. 2018. “Local‐Global Parcellation of the Human Cerebral Cortex From Intrinsic Functional Connectivity MRI.” Cerebral Cortex 28, no. 9: 3095–3114. 10.1093/cercor/bhx179. 28981612 PMC6095216

[hbm70268-bib-0030] Schmahmann, J. D. , X. Guell , C. J. Stoodley , and M. A. Halko . 2019. “The Theory and Neuroscience of Cerebellar Cognition.” Annual Review of Neuroscience 42: 337–364. 10.1146/annurev-neuro-070918-050258. 30939101

[hbm70268-bib-0031] Seghier, M. L. 2018. “Clustering of fMRI Data: The Elusive Optimal Number of Clusters.” PeerJ 6: e5416. 10.7717/peerj.5416. 30310731 PMC6173948

[hbm70268-bib-0032] Telesford, Q. K. , J. H. Burdette , and P. J. Laurienti . 2013. “An Exploration of Graph Metric Reproducibility in Complex Brain Networks.” Frontiers in Neuroscience 7: 67. 10.3389/fnins.2013.00067. 23717257 PMC3652292

[hbm70268-bib-0033] Thirion, B. , G. l. Varoquaux , E. Dohmatob , and J.‐B. Poline . 2014. “Which fMRI Clustering Gives Good Brain Parcellations?” Frontiers in Neuroscience 8: 1–13. 10.3389/fnins.2014.00167. 25071425 PMC4076743

[hbm70268-bib-0034] Thomas Yeo, B. T. , F. M. Krienen , J. Sepulcre , et al. 2011. “The Organization of the Human Cerebral Cortex Estimated by Intrinsic Functional Connectivity.” Journal of Neurophysiology 106, no. 3: 1125–1165. 10.1152/jn.00338.2011. 21653723 PMC3174820

[hbm70268-bib-0035] Varikuti, D. P. , F. Hoffstaedter , S. Genon , H. Schwender , A. T. Reid , and S. B. Eickhoff . 2016. “Resting‐State Test–Retest Reliability of a Priori Defined Canonical Networks Over Different Preprocessing Steps.” Brain Structure and Function 222, no. 3: 1447–1468. 10.1007/s00429-016-1286-x. 27550015 PMC5322256

[hbm70268-bib-0036] Wang, C. , J. Kipping , C. Bao , H. Ji , and A. Qiu . 2016. “Cerebellar Functional Parcellation Using Sparse Dictionary Learning Clustering.” Frontiers in Neuroscience 10: 188. 10.3389/fnins.2016.00188. 27199650 PMC4852537

[hbm70268-bib-0037] Wang, Y. , L. Zhu , Q. Zou , et al. 2018. “Frequency Dependent Hub Role of the Dorsal and Ventral Right Anterior Insula.” NeuroImage 165: 112–117. 28986206 10.1016/j.neuroimage.2017.10.004

[hbm70268-bib-0038] Xue, A. , R. Kong , Q. Yang , et al. 2021. “The Detailed Organization of the Human Cerebellum Estimated by Intrinsic Functional Connectivity Within the Individual.” Journal of Neurophysiology 125, no. 2: 358–384. 10.1152/jn.00561.2020. 33427596 PMC7948146

[hbm70268-bib-0039] Yan, C. G. , X. D. Wang , X. N. Zuo , and Y. F. Zang . 2016. “DPABI: Data Processing & Analysis for (Resting‐State) Brain Imaging.” Neuroinformatics 14, no. 3: 339–351. 10.1007/s12021-016-9299-4. 27075850

[hbm70268-bib-0040] Zhi, D. , M. King , C. R. Hernandez‐Castillo , and J. Diedrichsen . 2022. “Evaluating Brain Parcellations Using the Distance‐Controlled Boundary Coefficient.” Human Brain Mapping 43, no. 12: 3706–3720. 10.1002/hbm.25878. 35451538 PMC9294308

[hbm70268-bib-0041] Zuo, X. N. , and X. X. Xing . 2014. “Test‐Retest Reliabilities of Resting‐State FMRI Measurements in Human Brain Functional Connectomics: A Systems Neuroscience Perspective.” Neuroscience and Biobehavioral Reviews 45: 100–118. 10.1016/j.neubiorev.2014.05.009. 24875392

