# Supplementary material for: A Cerebellar Partitioning Method Using Spectral Clustering With Optimized Nonlinear Functional Connectivity
Source: Hum Brain Mapp. 2025 Jul 1;46(10):e70268. doi: 10.1002/hbm.70268 (PMC12214420; doi:10.1002/hbm.70268)
Supplement: Supplementary file 1 — Data S1. Supporting Information. [file HBM-46-e70268-s001.docx]

Supplementary Materials


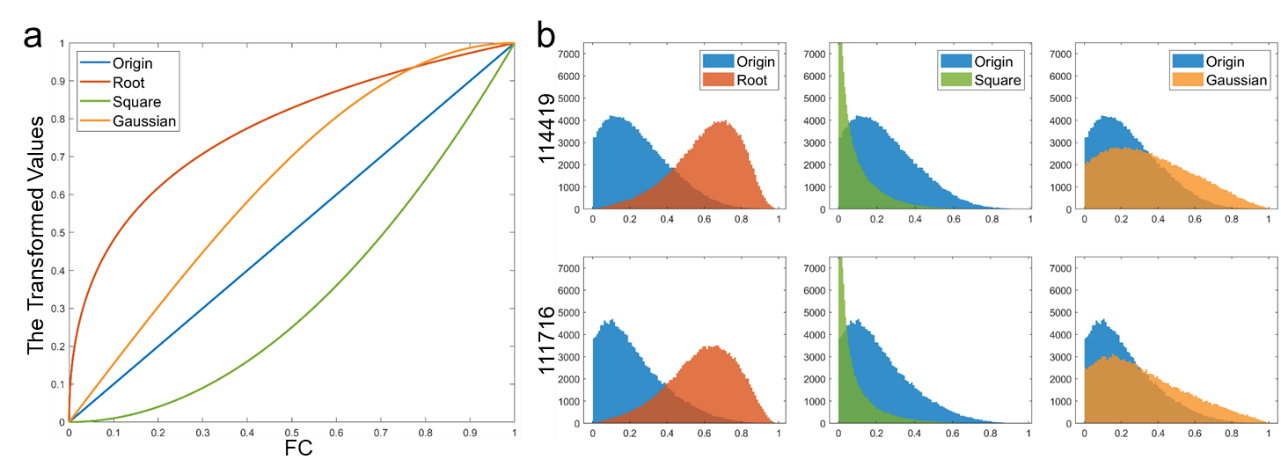


Supplementary Figure 1 Changes in FC distribution by different transformation functions. a shows the mapping between the three transformations and the original FC values, and b shows how the distribution of FC values is changed by the three transformations.


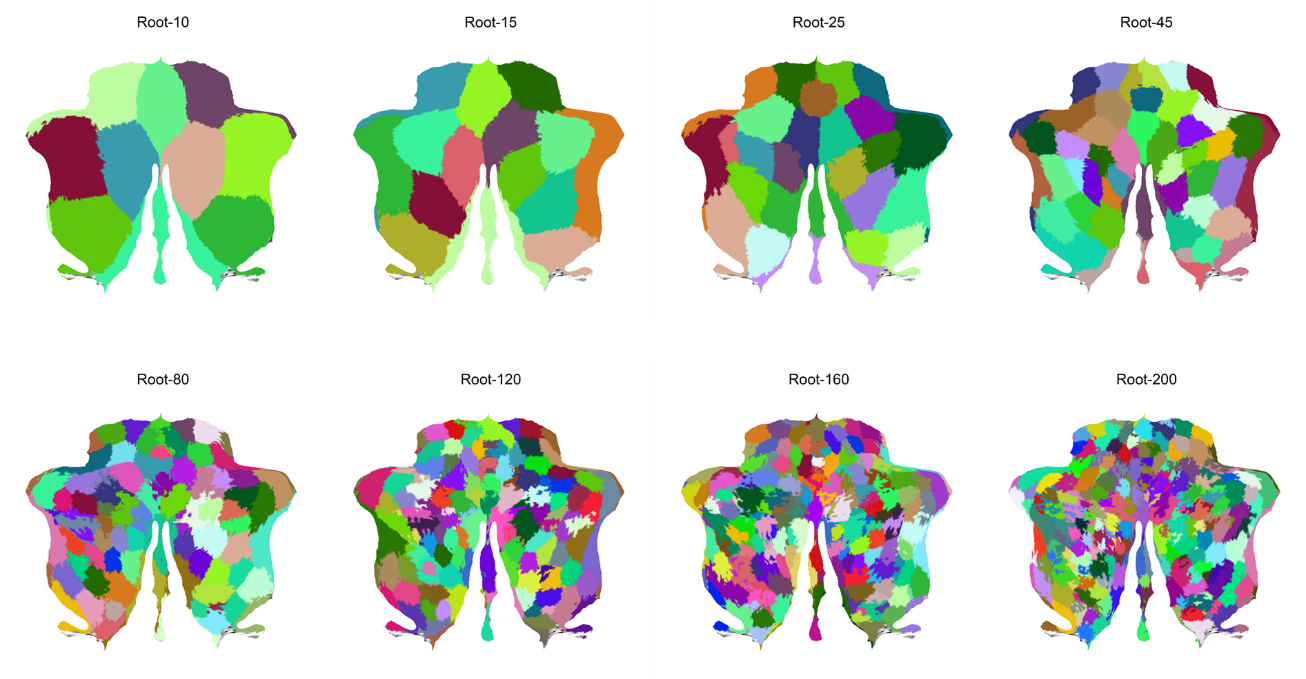


Supplementary Figure 2 Root cerebellar partitions with 10, 15, 25, 45, 80, 120, 160, 200 partitions planar visualization.
